# Measuring the effects of exercise in neuromuscular disorders: a systematic review and meta-analyses

**DOI:** 10.12688/wellcomeopenres.15825.1

**Published:** 2020-05-04

**Authors:** Renae J. Stefanetti, Alasdair Blain, Cecilia Jimenez-Moreno, Linda Errington, Yi Shiau Ng, Robert McFarland, Doug M. Turnbull, Jane Newman, Gráinne S Gorman

**Affiliations:** 1 Wellcome Centre for Mitochondrial Research, Newcastle University, Newcastle Upon Tyne, Tyne and Wear, NE2 4HH, UK; 2 Faculty of Medical Sciences, Newcastle University, Newcastle upon Tyne, Tyne and Wear, NE2 4HH, UK; 3 NIHR Newcastle Biomedical Research Centre, Newcastle upon Tyne Hospitals NHS Foundation Trust, Newcastle upon Tyne, Tyne and Wear, NE4 5PL, UK

**Keywords:** Neuromuscular, exercise training, aerobic, strength, functional exercise, muscle biopsies, minimal important clinical difference, outcome measures

## Abstract

**Background: **The benefit and safety of exercise training for patients with neuromuscular disorders (NMDs) has long been a contentious topic. This is, in part, due to recognised challenges associated with rare diseases including small and heterogenous patient populations. We performed a systematic review and meta-analyses to evaluate the effectiveness and safety of interventional exercise and establish minimal clinically important differences (MCID) in outcomes to facilitate clinical interpretation.

**Methods: **We searched six databases from inception to Mar 2018. Aerobic, strength, and combined (aerobic and strength) intervention were eligible. Meta-analyses compared outcomes at baseline with those after at least six weeks (before-after exercise within individuals). A further meta-analysis compared outcomes before-after exercise between groups (exercise training versus usual care). Disease heterogeneity was explored using a random effect model. This study was registered (PROSPERO, CRD42018102183). An interactive database was developed to facilitate full interrogations of data.

**Results: **We identified 130 articles describing 1,805 participants with 35 different forms of NMD. Of these studies, 76 were suitable for meta-analyses. Within group and between group meta-analyses detected an increase in peak aerobic capacity (p=0·04), and peak power (p=0·01). Six-minute walk test (p=0·04), sit-to-stand (STS) (repetitions) (p=0·03), STS (seconds) (p=0·04), rise from supine (p=0·008), SF-36 (p=0·0003), fatigue severity (p=<0·0001), citrate synthase (p=0·0002), central nuclei (p=0·04), type 1 (p=0·002) and type II muscle fibre area (p=0·003), were only able to detect change within group meta-analyses. Substantial *I* ^2^ statistic heterogeneity was revealed for STS (seconds) ( *I*²=58·5%; p=0·04) and citrate synthase ( *I*²=70·90%; p=0·002), otherwise heterogeneity for all outcomes was low. No study-related serious adverse events were reported nor significant increases in creatine kinase.

**Conclusions: **Exercise training in patients with NMDs appears to cause no harm across a range of outcomes. With the emergence of new therapeutic strategies, defining MCID is vital in informing future clinical trial design.

**Research in context**

**Evidence before this study**

It is increasingly recognised that exercise is beneficial as a therapeutic strategy in patients with neuromuscular disorders. However, systematic reviews and meta-analyses performed to date have only considered randomised or quasi-randomised trials, thereby drawing the conclusion that greater research is required.

**Added value of this study**

Our systematic review and meta-analysis is the broadest and most robust analysis, investigating a pooled meta-analysis of the most frequently utilised outcome measures used across exercise intervention studies in patients with neuromuscular disorders. The outcome measure database generated is intended to provide clinicians and researchers with a relevant, accessible, evidence-based tool to help direct and/or support exercise prescription and assessment.

**Implications of all the available evidence**

Utilising the most appropriate outcome measures is essential to generate meaningful and usable inferences to facilitate future research, and clinical practice. Health policy and guidelines, including increased specialised support to implement exercise therapy is required to translate these clinically significant evidence-based findings into clinical practice.

## Introduction

Neuromuscular disorders (NMDs) are a heterogeneous group of inherited or acquired disorders that impair skeletal muscle function. NMDs tend to be progressive, with many patients experiencing muscular weakness, fatigue and pain, resulting in reduced endurance and quality of life. As there is no cure for most NMDs, a primary aim of treatment is to improve or maintain function and mobility. Whether exercise is beneficial or deleterious in the case of NMD has been a debatable topic over the decades. However, the negative impacts of inactivity are well known; further deconditioning and exercise intolerance exacerbates disease symptoms, poorer health outcomes and co-morbidities ^1^. Despite this, patients with NMD (and some clinicians) are still cautious when it comes to engaging in physical activity.

Whilst numerous studies have investigated the influence of exercise training in NMD, the role of exercise as a therapeutic intervention is not fully appreciated nor widely implemented in clinical practice. Meta-analyses performed to date have evaluated the effect of defined exercise modalities in specific-NMD ^2– 7^. However, given their restriction to randomised control trials (RCTs) or quasi-randomised trials, these reviews have generally concluded that there is insufficient evidence to draw a meaningful conclusion of effectiveness.

Therefore, we aimed to undertake a comprehensive approach, inclusive of additional study designs such as single group trials (before-after) in order to elucidate the safety and efficacy of exercise training in NMD. The aim of the present meta-analysis was to combine exercise intervention data across cohorts of NMD to gain a more reliable estimate of the efficacy and safety of exercise training. First, we aimed to systematically identify the most frequently utilised outcomes in exercise intervention trials across key outcome domains. Second, we aimed to determine the appropriateness of these outcome measures via performing meta-analyses and lastly, we aimed to investigate the safety of exercise training in NMD.

## Methods

### Search strategy and selection criteria

In this systematic review and meta-analysis, we searched MEDLINE, Embase, Scopus, Web of Science, SportDiscus, and Cochrane Library for articles published between database inception and Mar 26, 2018. Reference lists of eligible studies and related reviews were hand searched to identify further studies. Search terms used in the MEDLINE search are included in the ( *Extended data*, Table 1, p 3) ^8^. To be eligible, articles had to assess exercise training with an intervention that was primary aerobic training (AET), strengthening/resistance training, or a combination thereof (aerobic and strength) for at least six weeks in patients with a clinically defined NMD ^9^. Articles with healthy control participants, non-exercise trained NMD patients (i.e. usual care), or without a comparative cohort were included. Only articles published in English were considered. Data from unpublished trial registries, abstracts, or conference proceedings were not included. Detailed study inclusion/exclusion criteria are provided in *Extended data* (p 4) ^8^. Two authors (RS and JN) screened study titles and abstracts. Discrepancies in the inclusion or exclusion of articles were reconciled through discussion. Articles with any study design were eligible for systematic review. However, case studies and case series reports were not included in the meta-analyses to ensure the most reliable comparisons ^10^.

Articles were omitted from meta-analysis if they did not report data in a format other than mean (standard deviation) or mean (standard error of the mean); corresponding authors were contacted wherever possible to obtain these data by use of individually tailored data forms. If an article reported outcome data at multiple time points, the duration containing the largest sample was selected for meta-analysis. When two or more time points included equal sample sizes, the data reported at the end of the intervention was selected, consistent with a relevant meta-analysis in the field ^7^. Meta-analysis fell into two distinct categories based on availability of data: within-group (before versus after exercise training) and between-groups (exercise training versus usual care). This review was done in accordance with the Preferred Reporting Items for Systematic Reviews and Meta-Analysis (PRISMA) guidelines (PRISMA checklist available in *Extended data*, p 100) ^8^. The protocol is available online at PROSPERO registration ID CRD42018102183.

The assortment of outcome measures followed a content analysis approach ^11^. Initially, outcomes were extracted verbatim and then grouped by hand (RS) across distinct outcome domains as they emerged. Four study investigators cross-checked outcome allocation, with conflicting opinions resolved through discussion. Thereafter, raw data from frequently utilised outcome measures that were deemed relevant in the application of clinical trials were extracted (RS) and checked for accuracy (JN).

### Data analysis

A data extraction template was developed to electronically record patient and study characteristics including sample size, study population, demographics, interventions, outcomes, and data for meta-analysis. Where duplicate articles of the same trial were identified, outcome data for meta-analysis was only included from the first publication ^12^. A restricted maximum likelihood (REML) random-effects model was used to compute pooled estimates of effect size and corresponding 95% confidence intervals (95% CI) ^12^. Effect size was calculated using Cohen’s *d*, with an effect size of 0.2 defined as small, 0.5 defined as moderate, and 0.8 defined as largest ^13^. Standardised mean differences (SMDs) with 95% CIs were calculated as the difference in means between groups divided by the pooled SD. The pooled SMDs were re-expressed in their original unit of measure to interpret the clinical relevance of the outcome measures that reached statistical significance. Outcome data was extracted as analysed or per-protocol because of the high attrition unrelated to the intervention (attrition rate is provided in *Extended data*, Table 6 and Table 7, p 38–42) ^8^. Risk of bias and study quality was assessed by two independent investigators using The Cochrane risk of bias tool ^14^ and an adapted NIH Quality Assessment Tool for Before-After (Pre-Post) Studies With No Control Group ^15^. Publication bias was assessed via funnel plots with trim and fill, and estimated using Egger’s regression intercept ^16^. The quality of evidence for outcomes was rated using the grading of recommendations assessment, development, and evaluation (GRADE) approach ^17^.

A minimum of four comparisons were used to ensure meaningfully pooled data. Heterogeneity was assessed using the *I* ^2^ statistic with *I* ^2^ > 75% considered substantial heterogeneity, and the chi-squared test ^10^ with a p value < 0.10 to define significant heterogeneity. We did pre-specified subgroup (≥ two studies) and sensitivity analyses to investigate interventional moderators, including training modality, interventional duration, and type of NMD. Additional sensitivity analyses were performed where appropriate ( *Extended data*, p 5) ^8^. Power statistics for paired t-tests determined the number of patients with NMDs that would be required to achieve various effect sizes before-after interventional trials ( *Extended data*, p 89) ^8^. All meta-analyses were done in R (version 3.4.) using the metafor package (version 2.0.0) ^18^. The study is registered with PROSPERO, number CRD42018102183.

## Results

**Figure 1.**
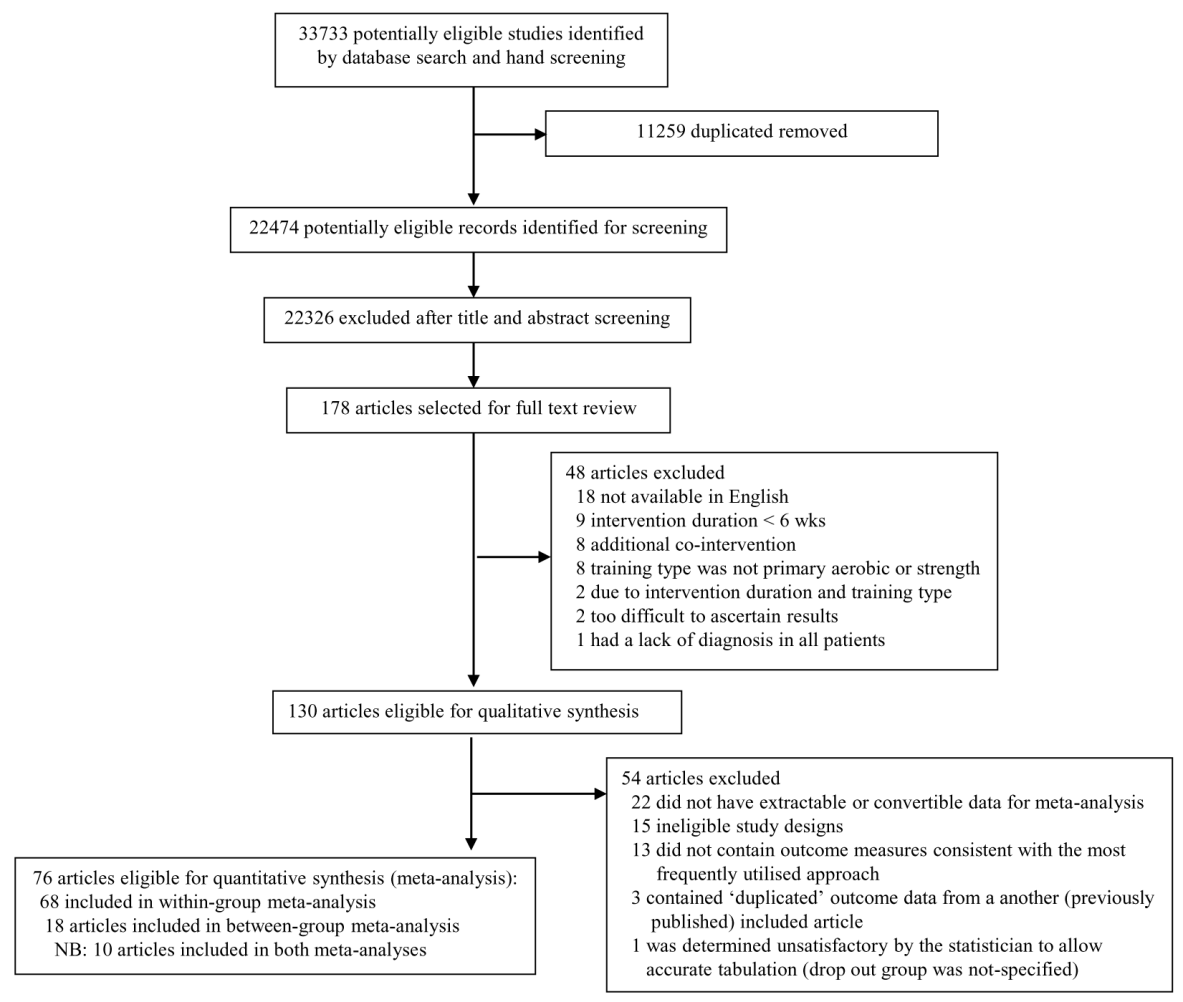
Flow chart of study selection.

In total, after removal of duplicates, we screened 22,474 abstracts, of which 178 were eligible for full-text review ( Figure 1). Of these, 130 articles with 1805 patients with NMD were deemed eligible for inclusion in the systematic review ( *Extended data*, Table 3, p 7, *Underlying data*, File 1) ^8^. The mean age of NMD patients that participated in an exercise training intervention was 45.4 ± 13.0 SD (range 6.5 to 67.3 years) of whom 41.9% were men ( *Underlying data*, File 4) ^8^. The most frequently utilised outcome measures (n=26) across key domains, including cardiopulmonary; muscle strength; functional capacity; activities of daily living (ADL); quality of life (QoL) and wellbeing; safety and pathophysiology, and muscle biopsy parameters were selected for meta-analysis. A total of 76 articles were included for meta-analysis, five of which included children. A total of 18 articles compared data between exercise training and usual care ( *Underlying data*, File 6) ^8^ and 68 articles compared within-group data before versus after exercise training ( *Underlying data*, File 7) ^8^. In total, 10 articles allowed both types of meta-analyses ( *Underlying data*, File 7) ^8^.

### Exercise training versus usual care

**Figure 2.**
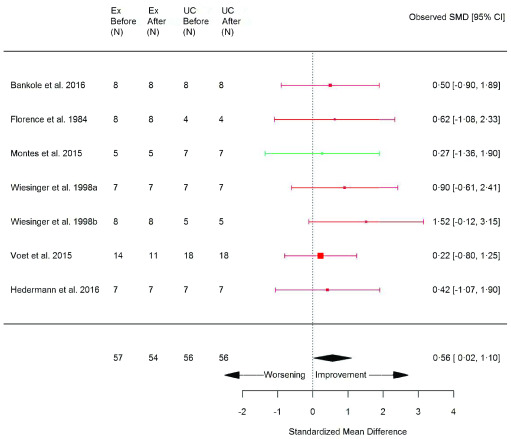
VO _2peak_ (ml/kg/min) (exercise training versus usual care). Random-effects meta-analysis of exercise-trained (Ex) compared to usual care (UC) on VO _2peak_ (ml/kg/min); pooled analysis of all trials using the final training intervention time point. Red denotes aerobic training; green, combined training.

**Figure 3.**
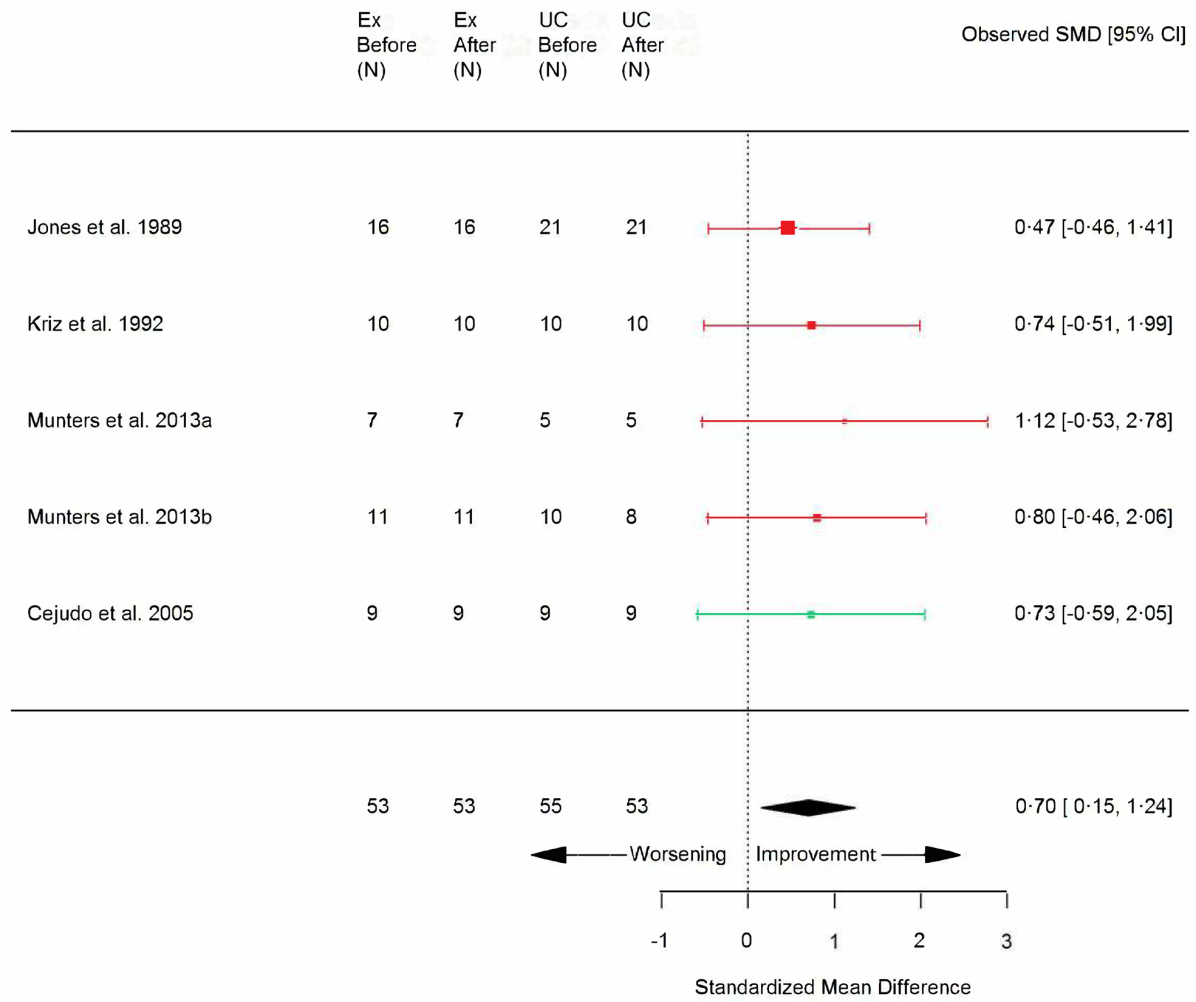
Peak power (watts) (exercise training versus usual care). Random-effects meta-analysis of exercise-trained (Ex) compared to usual care (UC) on peak power (watts); pooled analysis of all trials using the final training intervention time point. Red denotes aerobic training; green, combined training.

Articles for exercise training versus usual care meta-analysis were primarily RCTs (83%; n=15/18) ( *Underlying data*, File 6) ^8^. Exercise training demonstrated a significant improvement in peak aerobic capacity (VO _2peak_) (SMD 0.56 [95% CI 0.02, 1.10]; p=0.04) ( Figure 2) and peak power (Wpeak) (SMD 0.70 [95% CI 0.15, 1.24]; p=0.01) ( Figure 3) compared with usual care ( Table 1). However, no significant benefit in six-minute walk test (6MWT) or timed up and go (TUG) were noted ( Table 1; *Extended data*, Figure 12 and 13, respectively, p 47–48) ^8^. The only measure of QoL and wellbeing whereby data allowed meta-analysis between exercise and usual care was the vitality subscale of the Short Form 36 health survey (SF-36), whereby no difference was found ( Table 1; *Extended data*, Figure 14, p 48) ^8^. Muscle damage surrogate marker, creatine kinase (CK), was not significantly different in exercised patients compared with usual care (0.04 [95% CI -0.49, 0.58]; p=0.87) ( Table 1; *Extended data*, Figure 15, p 49) ^8^. The *I* ^2^ test statistic revealed no significant heterogeneity between articles comparing exercise versus usual care ( Table 1) though 10 of 18 articles were judged to be of unclear risk of bias ( *Extended data*, Table 6, p 38–39) ^8^. When performing subgroup analysis, VO _2peak_ and Wpeak also showed a significant improvement with exercise in the AET and inflammatory myopathies subgroups ( *Extended data*, Table 8, p 66 and Table 10, p 68, respectively) ^8^. Sensitivity analysis excluding an article including various types of NMD (Florence *et al.* 1984) did not alter the overall effect size of VO _2peak_, but reduced the statistical significance (p=0.06) ( *Extended data*, Table 16, p 76) ^8^. Due to the limited amount of data available, muscle strength and muscle biopsy outcomes were not possible to include for meta-analysis between exercise and usual care.

**Table 1.** Summary of exercise training versus usual care meta-analysis results for continuous variables. *P<0.05 compared to usual care. 6MWT, 6-minute walk test; SF-36, Short Form 36 health survey; TUG, timed up and go; VO _2peak_, maximal or peak aerobic capacity. Cohen’s *d* descriptor, M, moderate.

				Heterogeneity			Effect size		
Domain	Outcome measure	Studies (N)	Exercise Before/After (N)	Usual care Before/After (N)	*I ^2^*	P	SMD	95% CI	P	Cohen’s *d*
Cardiorespiratory	VO _2peak_ (ml/kg/min)	7	56/54	56/56	0	0.91	0.56	0.02, 1.10	0.04 *	M
	Peak power (watts)	5	53/53	53/53	0	0.97	0.70	0.15, 1.24	0.01 *	M
Functional capacity	6MWT (metres)	6	83/81	92/90	0	1.00	0.12	–0.31, 0.54	0.59	-
	TUG (seconds)	4	69/64	71/71	0	0.94	–0.07	–0.54, 0.41	0.78	-
	SF-36: Vitality (0-100)	4	66/66	67/67	0	0.86	0.11	–0.37, 0.59	0.65	-
Safety and pathophysiology	Creatine kinase (IU/l or U/l)	5	58/58	53/53	0	0.61	0.04	–0.49, 0.58	0.87	-

### Before versus after exercise training

**Figure 4.**
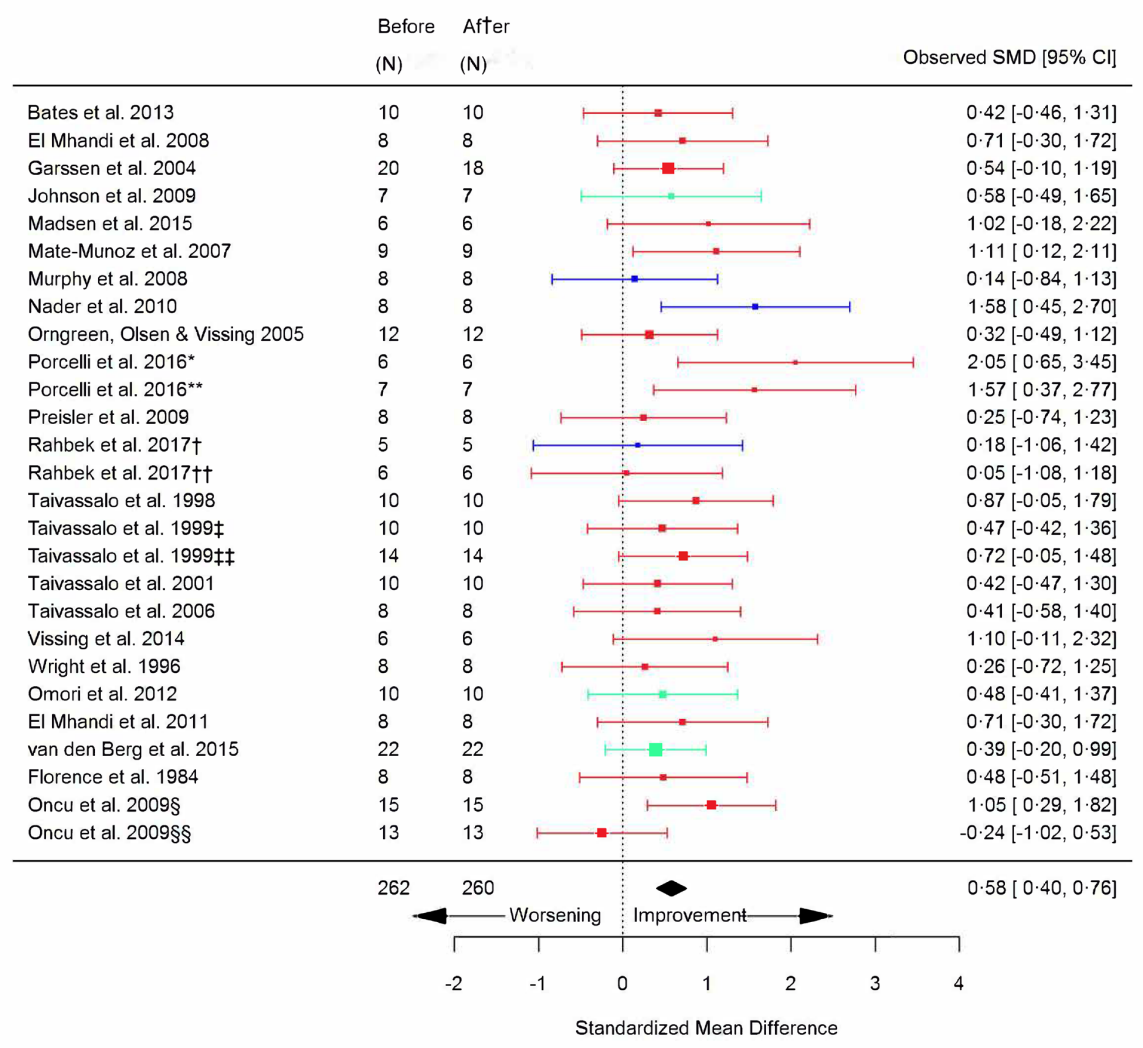
VO _2peak_ (ml/kg/min) (before versus after exercise training). Random-effects meta-analysis of exercise training on VO _2peak_ (ml/kg/min); pooled analysis of all trials using the final training intervention time point. Red denotes aerobic training; blue, strength training; green, combined training.*mitochondrial myopathies; **McArdle’s disease; ‡non-mitochondrial myopathies; §hospital-based training; §§home-based training.

**Figure 5.**
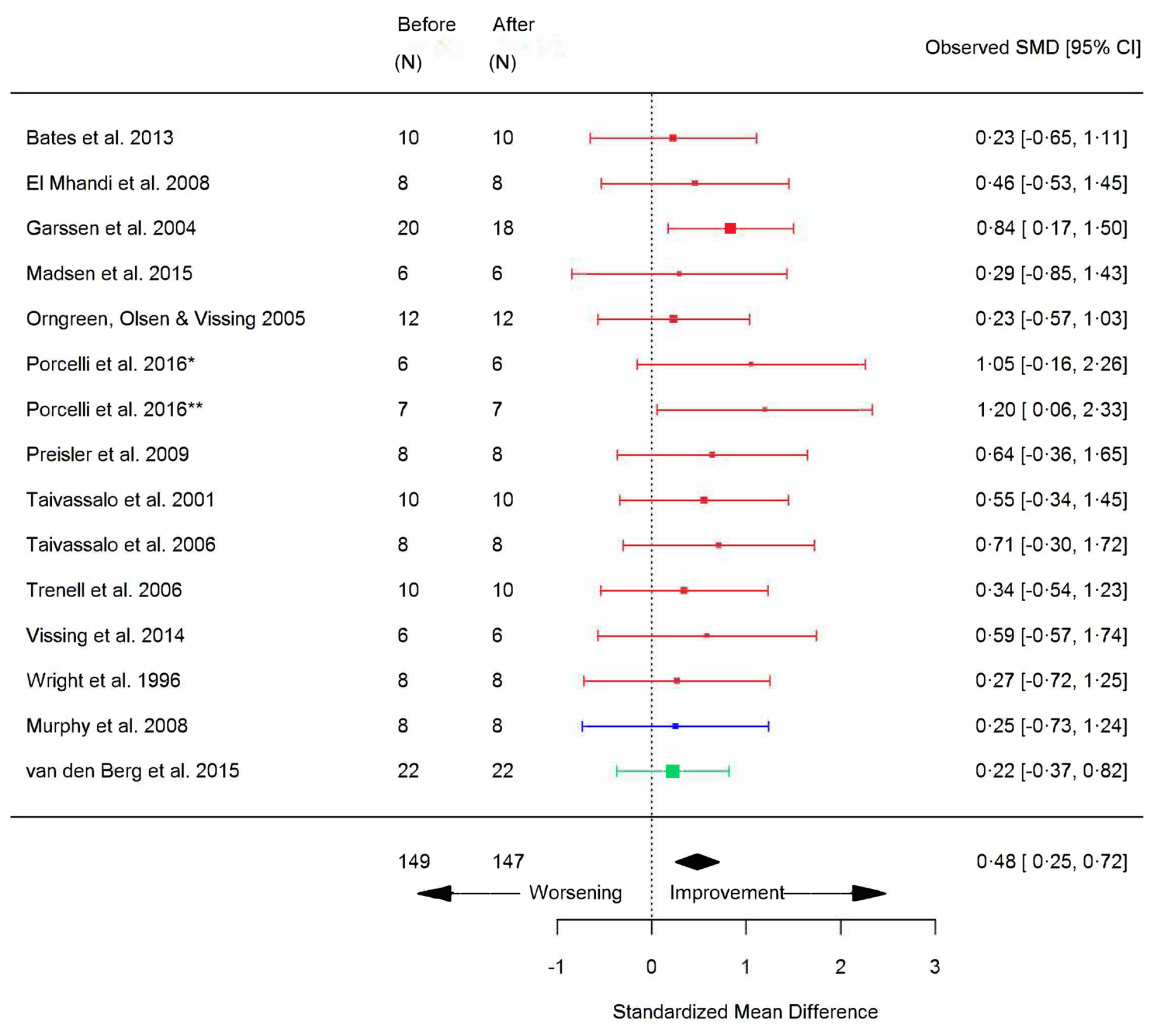
Peak power (watts) (before versus after exercise training). Random-effects meta-analysis of exercise training on Peak Power (watts); pooled analysis of all trials using the final training intervention time point. Red denotes aerobic training; blue, strength training; green, combined training.* mitochondrial myopathies; ** McArdle’s disease.

**Figure 6.**
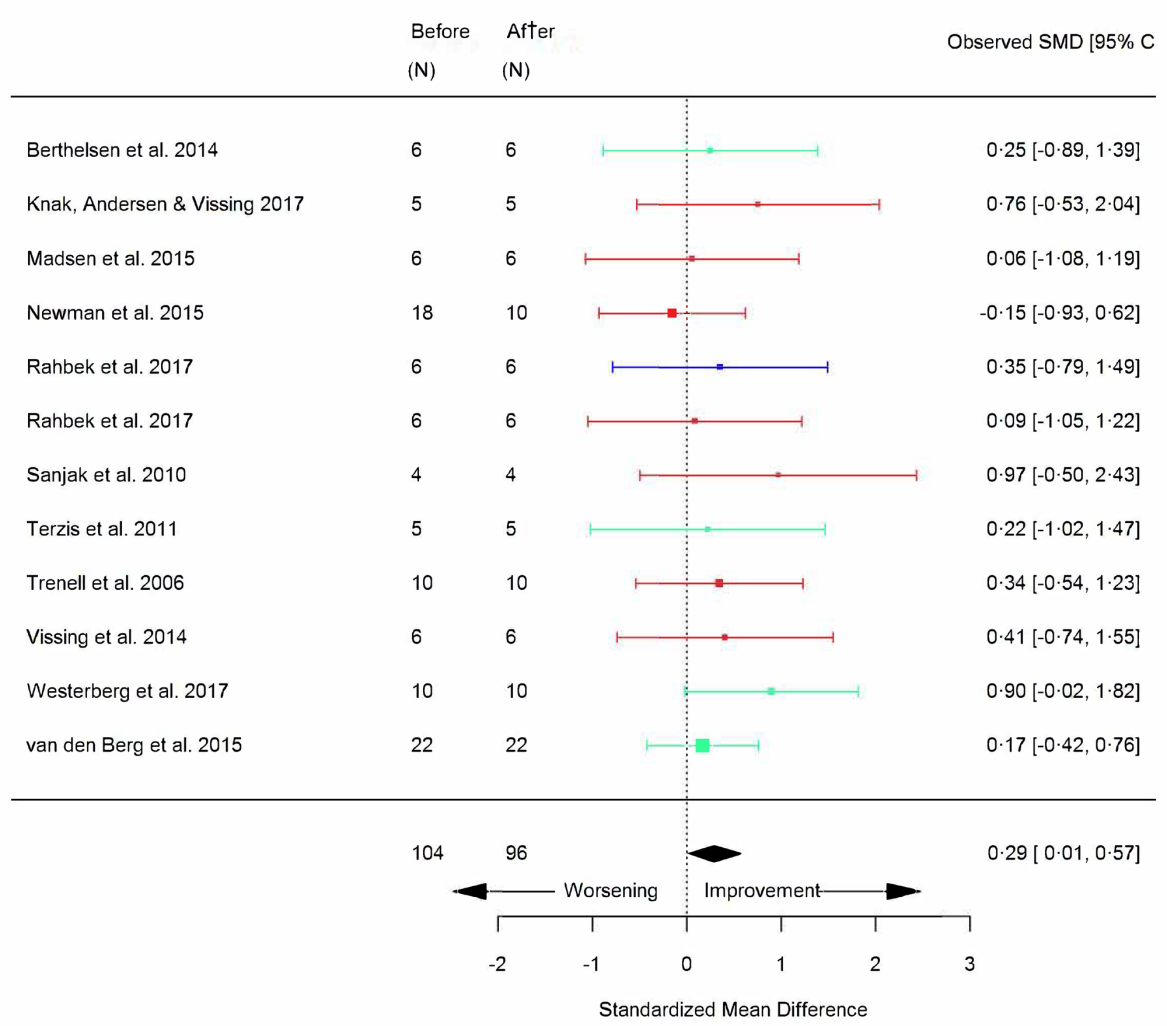
6MWT (metres) (before versus after exercise training). Random-effects meta-analysis of exercise training on 6MWT (metres); pooled analysis of all trials using the final training intervention time point. Red denotes aerobic training; blue, strength training; green, combined training.

**Figure 7.**
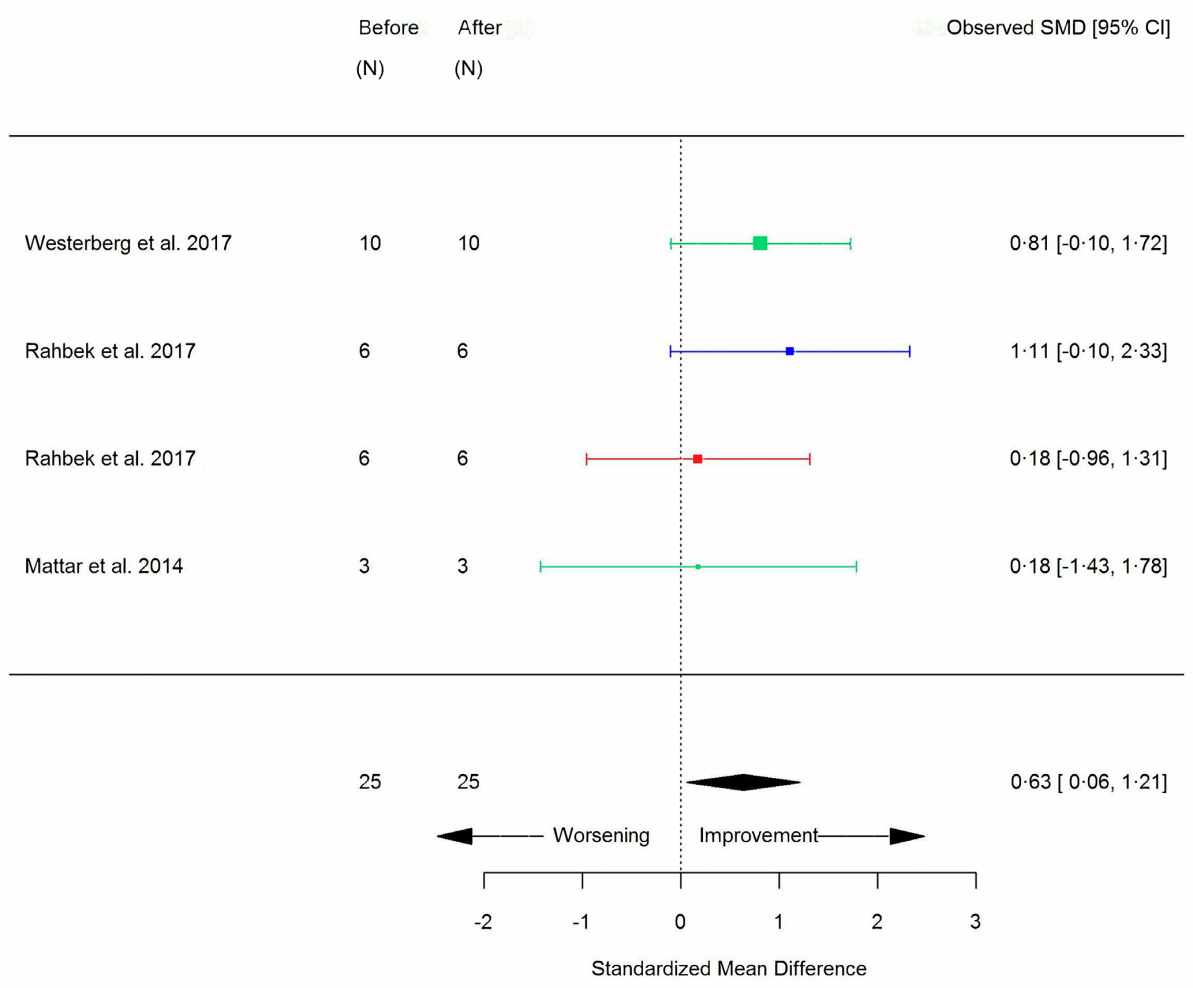
Sit-to-stand (STS) (repetitions) (before versus after exercise training). Random-effects meta-analysis of exercise training on STS (repetitions); pooled analysis of all trials using the final training intervention time point. Red denotes aerobic training; blue, strength training; green, combined training.

**Figure 8.**
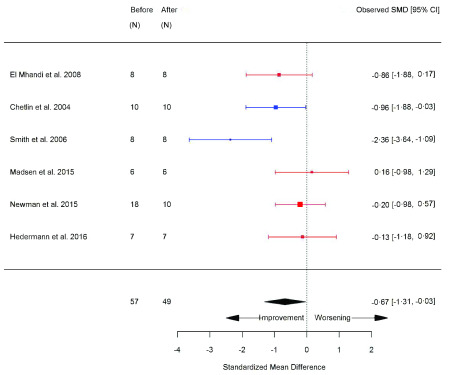
Sit-to-stand (STS) (seconds) (before versus after exercise training). Random-effects meta-analysis of exercise training on STS (seconds); pooled analysis of all trials using the final training intervention time point. Red denotes aerobic training; blue, strength training.

**Figure 9.**
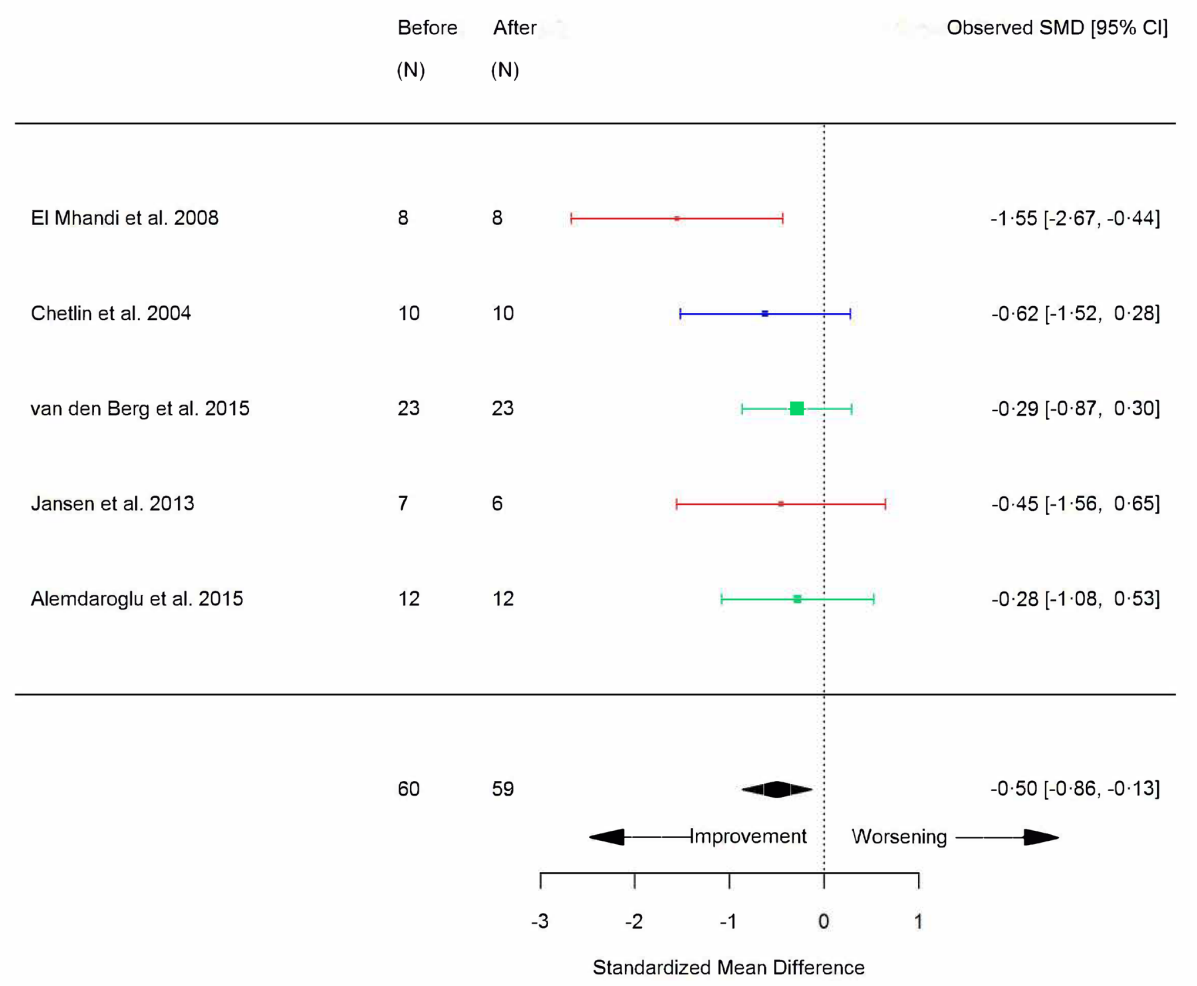
Rise from supine (seconds) (before versus after exercise training). Random-effects meta-analysis of exercise training on Rise from supine (seconds); pooled analysis of all trials using the final training intervention time point. Red denotes aerobic training; blue, strength training; green, combined training.

**Figure 10.**
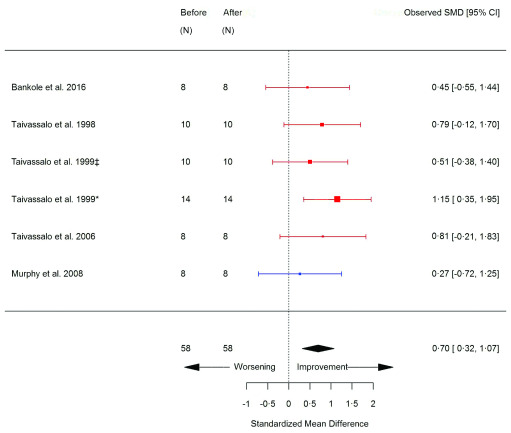
SF-36 (before versus after exercise training). Random-effects meta-analysis of exercise training on Short Form 36 health survey (SF-36); pooled analysis of all trials using the final training intervention time point. Red denotes aerobic training; blue, strength training. ‡non-mitochondrial myopathies; *mitochondrial myopathies.

**Figure 11.**
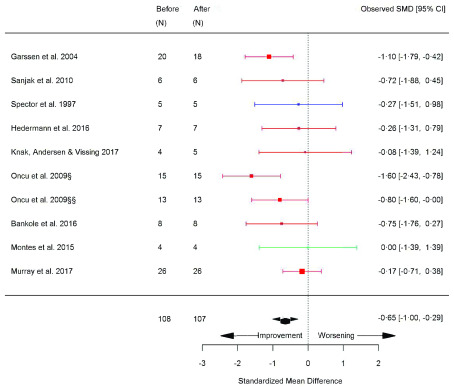
Fatigue severity scale (before versus after exercise training). Random-effects meta-analysis of exercise training on fatigue severity scale (FSS); pooled analysis of all trials using the final training intervention time point. Red denotes aerobic training; blue, strength training; green, combined training. §hospital-based training; §§home-based training.

**Figure 12.**
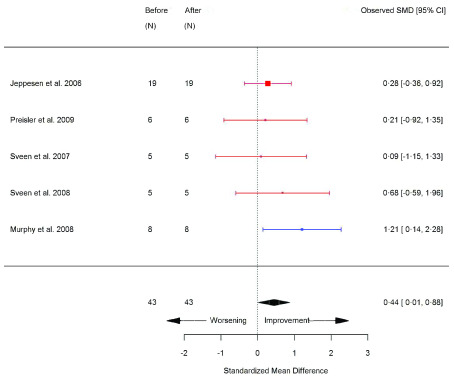
Central nuclei (%) (before versus after exercise training). Random-effects meta-analysis of exercise training on central nuclei (%); pooled analysis of all trials using the final training intervention time point. Red denotes aerobic training; blue, strength training.

**Figure 13.**
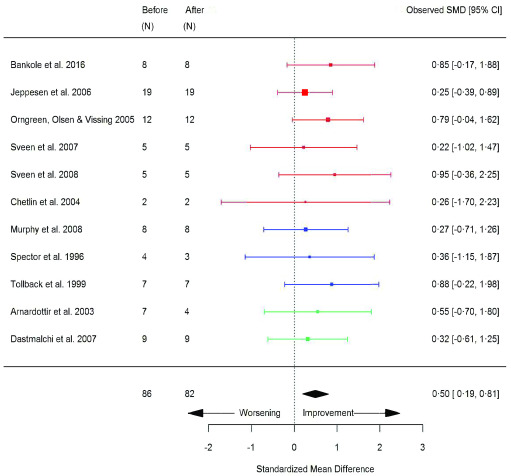
Type I fibre size area (µm or µm ^2^) (before versus after exercise training). Random-effects meta-analysis of exercise training on type I fibre size area; pooled analysis of all trials using the final training intervention time point. Red denotes aerobic training; blue, strength training; green, combined training.

**Figure 14.**
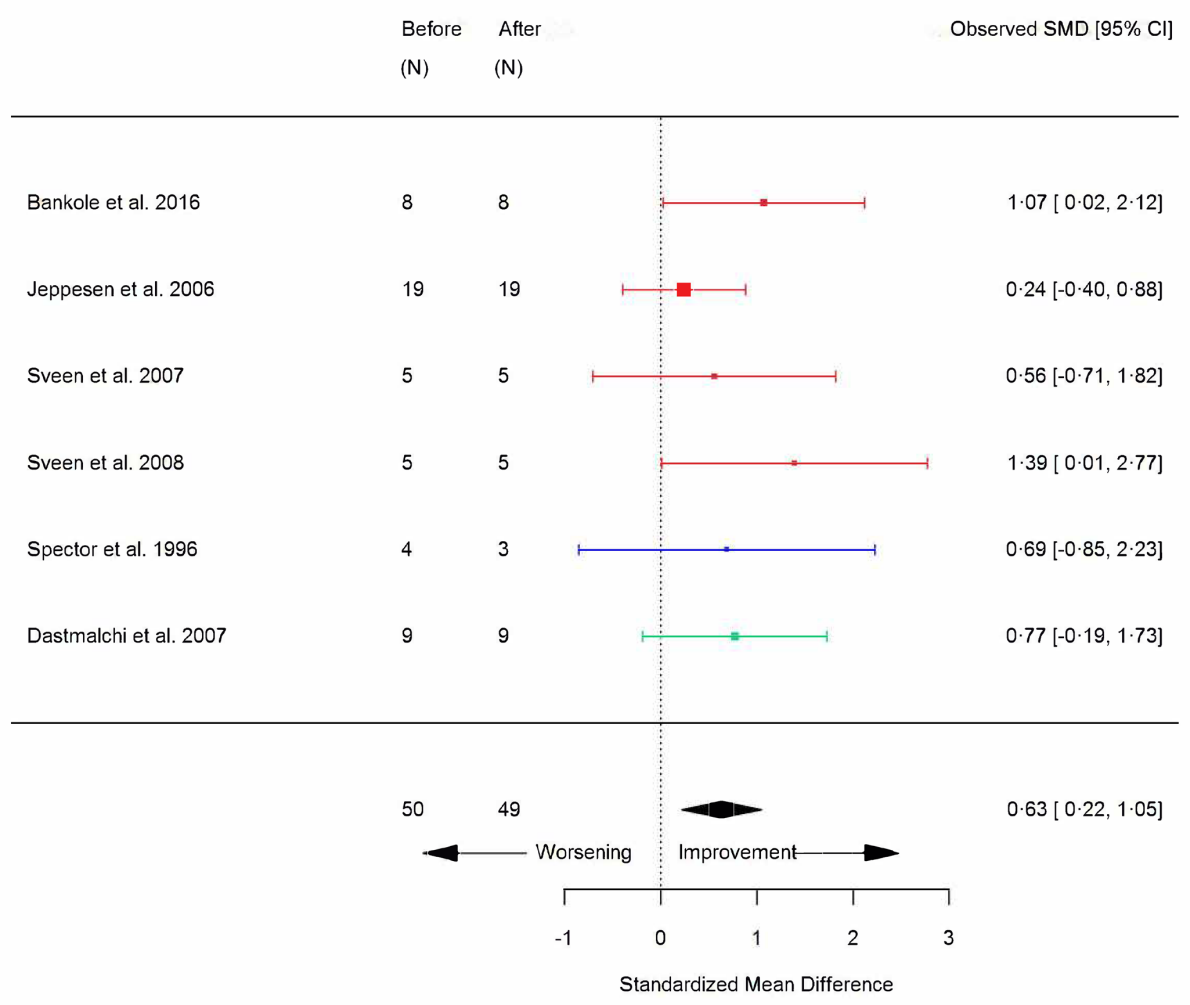
Type II fibre size area (µm ^2^) (before versus after exercise training). Random-effects meta-analysis of exercise training on type II fibre size area; pooled analysis of all trials using the final training intervention time point. Red denotes aerobic training; blue, strength training; green, combined training.

**Figure 15.**
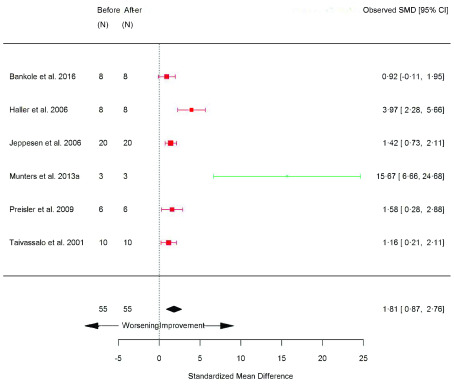
Citrate synthase (before versus after exercise training). Random-effects meta-analysis of exercise training on citrate synthase; pooled analysis of all trials using the final training intervention time point. Red denotes aerobic training; green, combined training.

Single-group (before-after exercise training) trials consisted of 69% (n=47/68) of all within-group meta-analyses ( *Underlying data*, File 7) ^8^. Meta-analysis was possible across all outcome domains ( Table 2). VO _2peak_ (SMD 0.58 [95% CI 0.40, 0.76]; p=<0.0001) ( Figure 4) and Wpeak (SMD 0.48 [95% CI 0.25, 0.72]; p=<0.0001) ( Figure 5) significantly increased after exercise ( Table 2). Although muscle strength outcomes did not differ significantly compared to before exercise ( Table 2), assessments of functional capacity largely improved, including; 6MWT (SMD 0.29 [95% CI 0.01, 0.57]; p=0.04) ( Figure 6), sit-to-stand (STS) (repetitions) (SMD 0.63 [95% CI 0.06, 1.21]; p=0.03) ( Figure 7), STS (seconds) (SMD -0.67 [95% CI -1.31, 0.03]; p=0.04) ( Figure 8), and rise from supine (SMD -0.50 [95% CI -0.87, -0.13]; p=0.008) ( Figure 9). SF-36 overall score significantly increased compared to before exercise (SMD 0.70 [95% CI 0.32, 1.07]; p=0.0003) ( Figure 10); whereas the SF-36 subscales were unchanged ( Table 2; *Extended data*, Figure 17–20, p51–53) ^8^. Skeletal muscle biopsy parameters significantly increased following exercise, including: type I fibre size area (SMD 0.50 [95% CI 0.19, 0.81]; p=0.002) ( Figure 13), type II fibre size area (SMD 0.63 [95% CI 0.22, 1.05]; p=0.003) ( Figure 14), central nuclei (a marker of muscle regeneration) (SMD 0.44 [95% CI 0.01, 0.88]; p=0.04) ( Figure 12), and citrate synthase (mitochondrial content marker) (SMD 1.81 [95% CI 0.87, 2.76]; p=0.0002) ( Figure 15), while fibre type distribution and capillary density did not change ( Table 2; *Extended data*, Figure 22–25, p 54–55) ^8^. Fatigue severity scale (FSS) was significantly reduced (SMD -0.65 [95% CI -1.01, -0.29]; p=<0.0001) ( Figure 11) and in accordance with the between-group meta-analysis, CK did not change before versus after exercise training (SMD -0.06 [95% CI -0.23, -0.11]; p=0.46) ( Table 2; *Extended data*, Figure 21, p 53) ^8^. A modified version of a standardized questionnaire was the most frequently used ADL outcome, albeit only used in AET studies. Patients self-rated a improvement in physical fatigue (16%; p=0.02), physical activity (32%; p=0.02), and walking distance (24%; p=0.04), while a non-statistical improvement was observed in muscle strength (63%; p=0.06) and endurance (72%; p=0.09) ( *Extended data*, Figure 10, p 45) ^8^. Importantly, a majority of patients did not self-rate a worsening in any ADLs (p≤0.01 to p≤0.0001, compared to self-rated improvement or no change) ( *Extended data*, Figure 11, p 46) ^8^.

**Table 2.** Summary of before versus after exercise training meta-analysis results for continuous variables. *P<0.05; **P<0.01; ***P<0.0001 compared to before exercise training. A number in parenthesis after the number of studies indicates individual comparisons in the analysis. 6MWT, 6-minute walk test; HHD, hand-held dynamometry; SF-36, Short Form 36 health survey; TUG, timed up and go; VO _2peak_, maximal or peak aerobic capacity. Cohen’s *d* descriptor, S, small; M, moderate; L, large.

			Patients	Heterogeneity		Effect size			
Domain	Outcome measure	Studies (N)	Before/After (N)	*I ^2^*	P	SMD	95% CI	P	Cohen’s *d*
Cardiorespiratory	VO _2peak_ (ml/kg/min)	23 (27)	262/260	0	0.64	0.58	0.40, 0.76	<0.0001 ***	M
	Peak power (watts)	14 (15)	149/147	0	0.97	0.48	0.25, 0.72	<0.0001 ***	M
Functional capacity	6MWT (metres)	11 (12)	104/96	0	0.94	0.29	0.01, 0.57	0.04 *	S
	STS (repetitions)	3 (4)	25/25	0	0.64	0.63	0.06, 1.21	0.03*	M
	STS (seconds)	6	57/49	58.05	0.04*	–0.50	–1.31, 0.03	0.04 *	M
	Rise from supine (seconds)	5	59/58	0	0.67	–0.50	–0.87, –0.13	0.008 **	M
	TUG (seconds)	4	37/29	0	0.97	–0.23	–0.72, 0.27	0.37	-
Quality of Life and wellbeing	SF-36 (0-100)	5 (6)	58/58	0	0.78	0.70	0.32, 1.07	0.0003 ***	M
	SF-36: Physical Function (0-100)	5	52/52	0	0.90	0.21	–0.18, 0.59	0.30	-
	SF-36: Vitality (0-100)	5	71/71	0	0.60	0.09	–0.24, 0.42	0.59	-
	SF-36: General Health (0-100)	4	48/48	0	0.64	0.23	–0.17, 0.64	0.26	-
	SF-36: Mental Health (0-100)	5	52/52	5.06	0.56	0.00	–0.40, 0.40	0.99	-
Safety and pathophysiology	Creatine kinase (IU/l or U/l)	29 (31)	278/277	0	1.00	–0.06	–0.23, 0.11	0.46	-
	Fatigue Severity Scale	9 (10)	108/107	33.56	0.18	–0.65	–1.01, –0.29	<0.0001 ***	M
Muscle biopsy parameters	Type I fibres (%)	8	70/68	0	0.63	0.10	–0.24, 0.43	0.58	-
	Type II fibres (%)	5	43/43	0	0.67	0.03	–0.39, 0.46	0.88	-
	Type IIa fibres (%)	4	35/33	0	0.38	–0.42	–0.90, 0.07	0.09	-
	Central nuclei (%)	5	43/43	0	0.58	0.44	0.01, 0.88	0.04 *	S
	Type I fibre area (um or um ^2^)	11	86/82	0	0.98	0.50	0.19, 0.81	0.002 **	M
	Type II fibre area (um ^2^)	6	50/49	2.04	0.64	0.63	0.22, 1.05	0.003 **	M
	Citrate synthase	6	55/55	70.9	0.002 **	1.81	0.87, 2.76	0.0002 ***	L
	Capillary density	5	47/47	45.89	0.12	0.04	–0.55, 0.64	0.88	-
Muscle strength	Isokinetic knee extension (peak torque)	11 (13)	127/123	0	1.00	0.22	–0.03, 0.47	0.09	-
	Isometric knee extension (HHD)	11	108/108	0	0.46	–0.03	–0.30, 0.24	0.90	-
	Isometric elbow flexion (HHD)	7	70/70	0	0.22	0.07	–0.26, 0.41	0.67	-
	Peak hand grip (grip dynamometer)	6	63/63	0	0.93	0.12	–0.23, 0.47	0.52	-

### Heterogeneity

We noted no evidence of heterogeneity with the exception of *I ^2^* statistically significant heterogeneity for STS (seconds) ( *I*²=58.05%; p=0.04) and citrate synthase ( *I*²=70.90%; p=0.002) ( Table 2). A total of 30 articles were judged as moderate quality and 26 were deemed to be high quality ( *Extended data*, Table 7, p 40–43) ^8^. Meta-analyses were virtually unaffected by sensitivity analysis when excluding articles judged to be of high or low risk of bias ( *Extended data*, Table 25–26, p 85–87) ^8^. Funnel plots suggested no evidence of publication bias ( *Extended data*, Figure 30–43, p 58–64) ^8^.

### Subgroup and sensitivity analyses

When performing subgroup analysis, AET retained significance in majority of outcomes with the exception of functional outcomes measures and central nuclei ( *Extended data*, Table 9, p 66–67). During subgroup analysis of the strength training group, STS was the only outcome reaching significance ( *Extended data*, Table 9, p 66) ^8^. VO _2peak_ remained statistically significant in disease subgroups of peripheral neuropathies, metabolic myopathies, inflammatory myopathies, and muscular dystrophies ( *Extended data*, Table 11, p 69) ^8^. Subgroup effects in muscle biopsy parameters occurred after training whereby citrate synthase increased in patients with metabolic myopathies and type II fibre size area increased in muscular dystrophies ( *Extended data*, Table 11, p 70) ^8^. Sensitivity analysis omitting strength training ^19^ from the before versus after exercise training meta-analysis on STS (repetitions), resulted in the effect being no longer statistically significant ( *Extended data*, Table 15, p 75) ^8^.

### Adverse effects and compliance

Likely study-related reasons for discontinuing from exercise training included joint or muscle pain/swelling [postpoliomyelitis syndrome (PPS; n=6 patients); ^20– 23^] musculoskeletal pain/discomfort in muscular dystrophies (n=6), ^24– 26^ and fatigue, predominantly in patients with non-specified congenital myopathies ^27^ (n=6/14). Mild muscle soreness or transient pain were frequently reported in the early training phase, particularly in patients with facioscapulohumeral muscular dystrophy (FSHD) ( *Extended data*, Table 28, p 90–97) ^8^. However, no study-related serious adverse events were reported. Alterations in training (i.e. postural adjustments, reduced intensity/volume/rest and frequency) facilitated training continuation ( *Extended data*, Table 28, p 90–97) ^8^. Encouragingly, the overall exercise training compliance was 87.4% ( *Extended data*, Figure 8, p 15) ^8^, further demonstrating a high tolerability of exercise training in patients with NMDs.

### Minimal clinically important differences (MCID) and power analysis

Re-expression of the pooled SMDs in their original units is shown in Table 3. Statistical power analysis of pooled data including our before versus after meta-analysis data revealed that in order to detect a Cohen’s *d* difference between two means ^13^ with a small effect, a sample size of 199 participants would be required, 34 participants for a moderate effect and 15 for a large effect (given an 80% power and 5% alpha) ( *Extended data*, Figure 44, p 89) ^8^.

**Table 3.** Re-expressed pooled SMDs using original unit of measure. A number in parenthesis after the number of studies indicates individual comparisons in the analysis. 6MWT, 6 minute-walk test; HHD, hand-held dynamometry; SF-36, Short Form 36 health survey; TUG, timed up and go; VO _2peak_, maximal or peak aerobic capacity. Cohen’s (1988) *d* was used to interpret the effect size, calculated via the standardised mean difference (SMD), with an effect size of 0.2 considered small, 0.5 moderate, and 0.8 large ^13^. For each outcome measure, the SD was pooled for the baseline data (i.e. before exercise training). Thereafter, the SMD was multiplied by the pooled SD to re-express the pooled effect for each outcome measure in its original units. To calculate the re-expressed pooled SMD in original units of measure, distribution based approaches were adapted from previous literature ^50^. Samsa (1999) advocated using a small effect size (0.2) to serve as an appropriate definition of a minimal clinical important difference (MCID) ^51^. To calculate the change scores equivalent to a small (0.2) magnitude, the pooled SD of the baseline scores for each outcome were multiplied by 0.2. Norman (1993) found that using 0.5 SD corresponded to a MCID; change scores were subsequently multiplied by 0.5 ^52^. Percentage change (95% CI) was calculated as the difference between the weighted baseline mean and the pooled re-expressed value. NB: for all continuous outcomes, only outcome measures that demonstrate a statistical significance were re-expressed, with the exception of citrate synthase due to substantial heterogeneity.

Exercise training versus usual care												
Domain	Outcome measure	Studies (N)	Patients (N)	SMD	Pooled SD	Cohen’s *d*	Pooled Re-expressed	% Change	95% CI	Small 0.2 SD	Moderate 0.5 SD	Large 0.8 SD
Cardio- respiratory	VO _2peak_ (ml/kg/min)	7	120	0.56	9.00	M	5.04	19.42	(16 to 22.85)	1.8	4.50	7.2
	Peak power (watts)	5	108	0.70	36.59	M	25.62	25.11	(20.17 to 30.06)	7.32	18.30	29.28
**Before versus after exercise training**												
Cardio- respiratory	VO _2peak_ (ml/kg/min)	23 (27)	262	0.58	6.01	M	3.49	16.38	(14.9 to 17.87)	1.20	3.01	4.81
	Peak power (watts)	14 (15)	149	0.48	53.16	M	25.52	19.52	(14.22 to 24.82)	10.63	26.58	42.53
Functional capacity	6MWT (metres)	11 (12)	104	0.29	111.08	S	32.21	6.99	(7.11 to 6.86)	22.22	55.54	88.86
	STS (repetitions)	3 (4)	25	0.63	4.34	M	2.74	20.00	(17.24 to 22.76)	0.87	2.18	3.48
	STS (seconds)	6	57	–0.50	6.84	M	-3.43	-31.77	(-41.65 to -21.88)	1.37	3.43	5.49
Quality of Life and	SF-36 (0-100)	5 (6)	58	0.70	11.06	M	7.74	20.71	(18.37 to 23.05)	2.21	5.53	8.85
Safety and patho- physiology	Fatigue Severity Scale	9 (10)	108	–0.65	3.50	M	-2.27	-23.80	(-15.19 to -32.4)	0.70	1.75	2.80
Muscle biopsy parameters	Central nuclei (%)	5	43	0.44	9.30	S	4.09	27.81	(-6.87 to 62.49)	1.86	4.65	7.44
	Type I fibre size area (um or um ^2^)	11	86/82	0.50	1321.32	M	660.66	15.68	(13.7 to 17.65)	264.26	660.66	1057.05
	Type II fibre size area (um ^2^)	6	50	0.63	1696.78	M	1068.97	21.77	(17.92 to 25.61)	339.36	848.39	1357.42

## Discussion

In an attempt to tackle the inherent challenges associated with rare diseases, our investigation is the first to conduct a data pooling analysis of the effect of exercise training across different forms of NMD. The high number of articles identified supports the notion that exercise is an intervention of intense interest across a variety of NMDs. However, a lack of high quality RCTs, to date, has limited meaningful conclusions being drawn on the effectiveness and safety of interventional exercise in NMD. Increasingly, non-randomised studies are now recognised to add value to this interpretation such as the inclusion of single group trials in addition to RCTs ^28– 30^, as adopted by this review.

Our systematic review revealed a vast number of outcome measures with little uniformity were available to assess exercise training across domains, highlighting a need to establish a core set of outcomes (and operational procedures) to facilitate data comparison. Our analysis identifies specific outcome measures that were able to identify MCIDs across a variety of NMDs. However, it should be noted that a limited number of studies included children, so the generalisability of our conclusions is limited to adults with NMD.

We have shown through analysing pooled data that when compared to usual care, NMD patients only have a significant improvement in peak aerobic capacity and peak power after exercise training (noting that the majority of interventions involved AET). When comparing before- and after-training, patients with NMD who undertook exercise training, demonstrated improvements in a multitude of domains including cardiorespiratory, functional capacity, QoL and well-being, and muscle biopsy parameters. Specifically, improvements in peak aerobic capacity were observed across NMD subgroups: peripheral neuropathies, metabolic myopathies inflammatory myopathies, and muscular dystrophies. As increased cardiopulmonary capacity is recognised, as a reliable predictor of health and longevity in the general population ^31^, this may have additive health benefits in these patient cohorts. Peripheral neuropathies and metabolic myopathies also increased Wpeak, possibly suggesting a superior response to training in these patient groups; although small sample sizes may limit this interpretation and significance in other NMD subgroups.

Muscle strength, as a measure of impairment as opposed to function, was assessed via traditional quantitative measures (i.e. hand-held myometry and isokinetic dynamometry), and lacked sensitivity to detect a significant change after exercise training. This is perhaps most disappointing when strength has been measured in almost half of all papers, and would intuitively be a relevant parameter to measure in diseases where muscle is the primary tissue affected. However, a lack of measurable change in strength assessments is not without precedence, potentially due to a lack of standardised protocols and specificity of training stimulus ^32^. Combined, these findings support the need for additional work to develop more sensitive and relevant methods of muscle strength assessment.

Functional outcome measures including STS, 6MWT and rise from supine significantly increased after training; whereas TUG failed to reach statistical significance. STS improved with strength training but not AET, revealing a higher contribution of strength to this outcome with a favourable responsiveness to change, suggesting this outcome may be a valuable surrogate functional marker of muscle strength. Upon sensitivity analysis excluding a single strength training study from the 6MWT before-after exercise-training meta-analysis (n=12), the overall effect was no longer statistically significant. This suggests that in patients with NMDs, 6MWT is not a pure measure (‘field test’) ^33^ of exercise capacity; but more an assessment of functional ability and likely impacted by other impairments such as muscle strength. Hence, we caution the conventional use (and interpretation) of the 6MWT ^34^ as an assessment of exercise capacity in this patient cohort. Furthermore, we have calculated that the pooled SMD in the 6MWT is 32 metres (6.99% [95% CI 6.86, 7.11]) before-after exercise training in patients with NMD. This correlated with previous established measures of MCID ^35– 37^.

Similar to AET-induced adaptations in healthy skeletal muscle, ^38^ a significant increase in muscle citrate synthase was observed in both pooled and subgroup analysis for AET. However, the substantial heterogeneity that was noted, was likely due to notorious methodological variability and differences in biopsy preparation ^39^. In contrast to normative adaptations to AET, no change in capillary density was observed, supporting the notion of an impaired vascular physiology, particularly in muscular dystrophies (4/5 meta-analysed studies) ^40^. Despite no change in muscle fibre composition, muscle fibre areas (type I and type II) significantly increased post interventional exercise. This observed increase was greatest in the AET and muscular dystrophies subgroups. These findings are in line with previous literature demonstrating that AET can be a stimulus for muscle fibre size increases in sedentary individuals, attributable to an increase in protein synthesis ^41^.

Few patient-reported outcome measures (PROMs) were sensitive enough to detect a significant change after interventional exercise. The universal applicability of the SF-36 as a QoL tool permitted analysis across various NMD subgroups. The overall SF-36 score was significant in the AET and metabolic myopathy subgroups. However, this was not the case for any of the SF-36 sub-scales. This was not surprising as sub-scales or single item measures have recognised limitations when evaluating interventions expected to impact ADL and QoL in a global manner ^42^, as in the case of exercise. As a recognised debilitating symptom in NMD, fatigue as measured by the FSS significantly improved following exercise. However, with the increasing interest from regulatory agencies to include PROMs in clinical trial design ^43^, further innovation in this area is needed.

There are also additional important research implications emerging from this study. The need to ensure that studies are adequately powered with a sample size sufficient to be able to detect a statistically significant difference in outcome measures is paramount; yet a recognised barrier ‘not least’ in rare disease clinical trial design. Of the 18 articles comparing exercise to usual care, only nine reported power and sample size calculations; and only four achieved the required sample size. This review now provides valuable data permitting sample size calculations to power future trials based on various outcome measures. In conjunction with this manuscript, we have developed an online resource ( http://www.newcastle-mitochondria.com/train-nmd/). This is an open access database of our meta-analyses, whereby each forest plot and associated data (NMD population and intervention modifiers) are available. Additionally, the systematic aggregation of all outcomes allows users to filter via various parameters (e.g. NMD type, intervention), providing an important evidence-based resource tool in both the clinical and research setting.

We acknowledge several limitations of this study and primarily highlight study design. Although conventionally other reviews only consider studies with low risk of bias ^2– 7^, adjusted meta-analyses have suggested that this may underestimate the benefits of a given intervention and hence we adopted this less conservative approach, in part due to the fundamental nature of exercise interventional studies in a rare disorder (namely small sample sizes and inadequate blinding of participants and/or assessor) ^44^. While we transformed effect sizes into original measurable units, it is known that this may lead to some inaccuracies ^45^. However, this interpretation is fundamental to deriving clinical meaning from the data. The articles included for meta-analysis consisted primarily of adults, therefore, the results may not be generalised to children with NMDs.

Our findings may also have wider clinical implications. Muscle weakness and wasting is now a recognised consequence of numerous other medical conditions, such as cancer, heart disease, and ageing (termed sarcopenia) ^46, 47^. Undoubtedly, a lack of exercise and deconditioning is a significant risk factor for premature death worldwide ^48, 49^. Implementation of accessible exercise regimes for patients with both primary and secondary muscle diseases will require adoption of new policies in multiple sectors. Initiatives to enhance personalised supervised exercise prescription in conjunction with greater utilisation of clinical specialists is required to translate these clinically significant evidence-based results into clinical care.

In conclusion, exercise training appears to be safe and effective in all forms of adult NMD with limited efficacy data in paediatric cases. Crucially, we have also been able to determine MCIDs that may inform future clinical trial design, in an era of rapidly emerging therapeutic strategies.

## Data availability

### Underlying data

Code Ocean: Measuring the effects of exercise in neuromuscular disorders: a systematic review and meta-analyses. https://doi.org/10.24433/CO.9997621.v2 ^8^.

Files 1 to 7 include raw data files for study details:

Files 1, 2, and 3 include tabulation of study populations (overall, exercised trained and usual care, respectively) for patients with NMD included for systematic review. Data is provided per included article and classified according to specific NMD.

1. Overall clinical population tabulation. Underlying data for Table 2, *Extended data*.

2. Exercise training tabulation. Underlying data for Table 3, *Extended data*.

3. Usual care tabulation. Underlying data for Table 3, *Extended data*.

File 4 includes tabulation of study details (gender, age) for all articles included for systematic review. Data is provided per included article for exercise trained and usual care patients with NMD.

4. Demographics tabulation. Underlying data for Table 5, *Extended data.*

File 5 includes tabulation of study design for all articles included for systematic review. File 5 also included tabulation of reasons articles were excluded from meta-analysis.

5. Study design_systematic review. Underlying data for Table 4, *Extended data* and part of Figure 1 (flow chart of study selection).

File 6 and 7 include tabulation of study design for respective meta-analyses. Outcome measures for meta-analysis are also tabulated via each included article.

6. Study design_outcome measures_meta-analysis 1 (exercise vs. usual care meta-analysis).

7. Study design_outcome measures_meta-analysis 2 (before vs. after exercise training meta-analysis).

File 8 to 12 include raw data files for intervention details:

File 8 includes tabulation of intervention setting and intervention supervision for all articles included for systematic review.

8. Setting and Supervision. Underlying data for Figure 1 and 2, respectively, *Extended data.*

File 9, 10, and 11 includes tabulation of interventional details for aerobic training, strength training and combined training, respectively.

9. Aerobic training details. Underlying data for Figure 3 to 7, *Extended Data.*

10. Strength training details. Underlying data for Figure 3 to 7, *Extended Data*

11. Combined training details. Underlying data for Figure 3 to 7, *Extended Data*

File 12 includes the linked spreadsheet workbook for the study interventional summary details.

Graph data for study intervention details Underlying (summary data) for Figure 1 to 7, *Extended Data*

### Extended data

Code Ocean: Measuring the effects of exercise in neuromuscular disorders: a systematic review and meta-analyses. https://doi.org/10.24433/CO.9997621.v2 ^8^.

The ‘data’ folder also presents the data and output for meta-analysis of three datasets related to studies of the effects of exercise in neuromuscular disorders (CSV format):

• ADL. Activities of Daily Living (ADL) meta-analysis data.
• Ex.vs.NonEx. Exercise vs Usual Care meta-analysis data.
• Pre.Post. Before and After exercise training meta-analysis data.

The ‘data’ folder also contains the Extended data.docx file (word document), providing the following:

• Search Strategy (Table 1).
• Summary details for systematic review; including neuromuscular disorders (NMD) (Table 2), study population groups (Table 3), and study designs (Table 4).
• Study interventional summary details (including setting, supervision, duration, frequency, session time, and compliance) (Figure 1–8) .
• Study details summary per article (including population group, demographics and intervention details) (Table 5).
• Study quality (Table 6 and Table 7).
• Ordinal activities of daily living (ADL) proportion data and forest plots (Figure 9–11).
• Forest plots of outcomes with no statistical significance (Figure 12–29).
• Funnel plots (Figure 30–43).
• Subgroup analysis (Table 8-13) and sensitivity analysis (Table 14–26).
Sample size calculation (before versus after exercise training) for future interventional trials (Figure 44).
• GRADE summary of findings (Table 27).
• Adverse events (Table 28).
• Excluded full text articles (Table 29).

The ‘code’ folder contains the R code used in the meta-analyses and to create funnel and forest plots.

### Reporting guidelines

Code Ocean: PRISMA checklist for ‘Measuring the effects of exercise in neuromuscular disorders: a systematic review and meta-analyses’ is available in Extended data.docx (page 100). https://doi.org/10.24433/CO.9997621.v2 ^8^.

Data are available under the terms of the Creative Commons Attribution 4.0 International license (CC-BY 4.0).

Code is available under the terms of the MIT License.

Exercise Training in Neuromuscular Disorders (NMD) Outcome Measures Database

Additionally, all meta-analysis and systematic review outcome data are available in an interactive database: ( http://www.newcastle-mitochondria.com/train-nmd/).

The 'Meta Analysis Results' tab presents an interactive graph of the key results from all the meta-analyses. The accompanying table provides the key statistics from each analysis along with links to more detailed data from the individual studies and the forest plot of the study and overall effect sizes.

The 'Systematic Review Database' tab presents information on individual studies within each outcome domain. The user can select subsets of these domains according to a variety of criteria. There is then the option to download more information on these defined subsets as a CSV file.
