# Supplementary material for: Measuring the effects of exercise in neuromuscular disorders: a systematic review and meta-analyses
Source: Wellcome Open Res. 2020 May 4;5:84. [Version 1] doi: 10.12688/wellcomeopenres.15825.1 (PMC7331112; doi:10.12688/wellcomeopenres.15825.1)
Supplement: Peer review history

# Peer review: Reviewer response for version 1

**DOI:** 10.21956/wellcomeopenres.17357.r38611

**Published:** 2020-07-01

**Authors:** Gita Ramdharry

**Affiliations:** 1 Queen Square Centre for Neuromuscular Diseases, University College London, London, UK; 2 National Hospital for Neurology and Neurosurgery, UCLH NHS Trust, London, UK

This is an excellent study conducted methodically and with rigour by an internationally renowned group in this field. The results are in depth but presently clearly with excellent interpretation of these findings to future exercise research in NMDs and also extrapolating to practise. 

 I have a small number of minor queries relating to the rationale for the work and interpretation of the results.

1. You state in the introduction that meta-analyses restricted to RCTs have not drawn meaningful conclusion of effectiveness. Could you go as far as to critiquing this method as being limited for this type of research? It is touched upon in the discussion, but to point out the implication of the limitations of the traditional meta-analysis would strengthen the rationale for your approach at the outset.
2. The lack of effect on muscle strength parameters. I agree with your point on the standardisation and specificity of training stimulus as a potential barrier to measurable change. Some may also ask if the underlying muscle disease limits the potential for change. This has been suggested in the past by some colleagues and could be explored here.
3. The muscle biopsy results reassuringly demonstrate that there is an increase in muscle fibre area/size. How do the authors explain this in relation to the lack of change in muscle strength when we assume that the two are correlated (as seen in normal data)? Does this imply that there isn't functional carry over, or is there something about the testing protocols for dynamometry that still leads to intrasubject variability due to additional factors. Always a risk where there is a voluntary element. 

Are the rationale for, and objectives of, the Systematic Review clearly stated?

Yes

Is the statistical analysis and its interpretation appropriate?

Yes

Are sufficient details of the methods and analysis provided to allow replication by others?

Yes

Are the conclusions drawn adequately supported by the results presented in the review?

Yes

Reviewer Expertise:

Rehabilitation interventions for neuromuscular diseases

I confirm that I have read this submission and believe that I have an appropriate level of expertise to confirm that it is of an acceptable scientific standard.

# Peer review: Reviewer response for version 1

**DOI:** 10.21956/wellcomeopenres.17357.r38613

**Published:** 2020-06-22

**Authors:** Mark Tarnopolsky

**Affiliations:** 1 Department of Pediatrics, Division of Neuromuscular and Neurometabolic Disorders, McMaster University Children's Hospital, Hamilton, ON, Canada

The authors have done an outstanding job of collating and evaluating the literature regarding the efficacy of exercise interventions in patients with NMD. As the authors have pointed out, there has been a lack of consensus regarding this treatment modality in this group due to small sample sizes in the studies. The power of the meta-analysis is very clear in that the confidence intervals for many of the outcome metrics from individual studies overlap 0, yet most of the important outcomes are clearly significant when all of the data is considered. Overall, this is a huge advancement to the field and will no doubt influence care.

 General comments:

1. As the authors indicate the conditions that are considered NMDs are also quite broad. It would be of interest to have a table to show which categories of NMDs have been evaluated. This would give more confidence knowing that a particular group was included in the analysis for individual exercise prescription. Perhaps Major groups such as 1. Metabolic myopathy - sub-group - mitochondrial (Taivassalo, etc.) McArdle, etc.; 2. Muscle dystrophy - Duchenne, LGMD, FSHD (Vissing, etc.) etc; 3. Inflammatory - IBM (Spector study, etc.). It would be nice to discuss what groups have not been studied yet for future research considerations (i.e., more in SMA given the advancements in Rx)
2. Have the authors considered looking at the age groupings - my sense is that most of the pediatric stuff is boys with DMD?

Are the rationale for, and objectives of, the Systematic Review clearly stated?

Yes

Is the statistical analysis and its interpretation appropriate?

Yes

Are sufficient details of the methods and analysis provided to allow replication by others?

Yes

Are the conclusions drawn adequately supported by the results presented in the review?

Yes

Reviewer Expertise:

Muscle disorders, mitochondrial disease, Pompe disease, metabolic glycogenoses, neurogenetics, aging, exercise physiology

I confirm that I have read this submission and believe that I have an appropriate level of expertise to confirm that it is of an acceptable scientific standard.
